# Association Between Methylmalonic Acid and Cognition: A Systematic Review and Meta-Analysis

**DOI:** 10.3389/fped.2022.901956

**Published:** 2022-06-29

**Authors:** Chao Wang, Ying Zhang, Jianbo Shu, Chunyu Gu, Yuping Yu, Wei Liu

**Affiliations:** ^1^Tianjin Pediatric Research Institute, Tianjin Children’s Hospital, Children’s Hospital of Tianjin University, Tianjin, China; ^2^Tianjin Key Laboratory of Birth Defects for Prevention and Treatment, Tianjin, China; ^3^Tianjin Children’s Hospital, Children’s Hospital of Tianjin University, Tianjin, China

**Keywords:** methylmalonic acid (MMA), cognition, meta-analysis, vitamin B12, dementia

## Abstract

**Background:**

Methylmalonic acid (MMA) is an intermediate metabolite of human body. The content of MMA in the blood of healthy people is very low, and its concentration will increase in some diseases and elderly people. Recent studies have shown that MMA has a variety of biological functions. The correlation between MMA and cognition, one of the important functions of the nervous system, is still uncertain.

**Objective:**

Meta-analyses were performed to assess whether elevated MMA was associated with the risk of cognitive decline.

**Materials and Methods:**

Cross-sectional studies, randomized controlled studies, and case-control studies on the relationship between MMA and cognition were obtained by searching PubMed, Web of Science, EMBASE, ProQuest, WANFANG MED ONLINE, China National Knowledge Infrastructure (CNKI) and Chongqing VIP until May 2022. Two researchers independently selected studies according to inclusion and exclusion criteria, evaluated study quality and extracted data. Meta-analyses were performed using Review Manager 5.4 software. The sensitivity analysis of meta-analysis was performed by One by one exclusion method.

**Results:**

A total of 11 studies were included, including six cross-sectional studies, two randomized controlled studies, and three case-control studies, with a sample of 16,533 subjects. Meta-analysis showed that there was no significant difference in cognitive level between high-level MMA subjects and low-level MMA subjects in the general population [SMD = −2.19, 95% CI (−4.76 ∼ 0.38), *Z* = 1.67, *P* = 0.09]. In the population supplemented with VitB12, the increase of MMA level caused by VitB12 supplementation was not related to the change of cognition [SMD = 0.32, 95% CI (−0.19 ∼ 0.84) *z* = 1.22, *P* = 0.22]. There was also no significant difference in MMA levels between patients with dementia and the control group [WMD = 20.89, 95% CI (−5.13 ∼ 46.92), *z* = 1.57, *P* = 0.12].

**Conclusion:**

In the general population, whether VitB12 is supplemented or not, there is no correlation between the increase of MMA level and the decrease of cognitive level. In dementia diseases, the level of MMA did not change significantly. High levels of MMA may not be a risk factor for cognitive impairment. The exact relationship between MMA and cognition needs further research.

**Systematic Review Registration:**

[https://www.crd.york.ac.uk/prospero/display_record.php?ID=CRD42021266310], identifier [CRD42021266310].

## Introduction

Cognitive impairment is one of the common manifestations of abnormal development or dysfunction of the nervous system. It is often seen in neurological, mental and elderly diseases, such as depression, neurasthenia and Alzheimer’s disease. Vitamin B12 (VitB12) has long been considered as a nutrient necessary for the development and functional maintenance of the nervous system. A variety of evidence-based medicine research results show that the lack of VitB12 can lead to a variety of neuropsychiatric diseases, including the decline of cognitive level (1).

In addition to VitB12 itself, methylmalonic acid (MMA), a binary acid in VitB12 related metabolic pathway, is the most commonly used marker in the medical detection of human VitB12, even more than VitB12 itself, this is because the serum VitB12 level with a certain lag is not consistent with the intracellular VitB12 state, while MMA can more quickly reflect the real demand state of human body for VitB12 (2, 3). In previous studies, MMA exists only as a metabolite or marker, and there are few research on the function of MMA itself. However, the latest researches show that MMA may have important biological functions. For example, one study has confirmed with sufficient and detailed evidence that MMA gradually accumulated with aging can induce transcriptional reprogramming by promoting SRY-box transcription factor 4 (SOX4) gene expression and promote tumor progression and invasion (4). Metabolism of cobalamin associated B protein (MMAB) can inversely regulate cholesterol metabolic balance through MMA (5). MMA can also damage the respiration of SH-SY5Y cells and reduce the expression of a variety of differentiation markers (6). The association between VitB12 and cognitive function has been proven in many studies, in some of which serum MMA completely represented VitB12 levels (7). Therefore, it cannot be ruled out that MMA is one of the factors that really play a role. In the research on the relationship between VitB12 and cognition, MMA was also detected as one of a variety of VitB12 markers, some of which also proved that single factor MMA was related to the decline of cognitive level (2), while some other study results showed that there was no correlation between them (8). Previous meta-analysis showed that total serum homocysteine (tHcy), another important marker of VitB12, was positively correlated with the incidence of dementia (9). The aim of this systematic review and meta-analysis was to evaluate the influence of MMA as a single factor on cognition, and draw a more objective conclusion.

## Materials and Methods

The review and meta-analysis were conducted according to the Preferred Reporting Items for Systematic reviews and Meta-Analyses (PRISMA) (10). Table 1 shows the Participants, Intervention, Comparison, Outcome and Study design (PICOS) criteria used to define the research question. The study was registered at the International Prospective Register of Systematic Reviews (PROSPERO) (ID: CRD42021266310).

**TABLE 1:** PICOS criteria for inclusion and exclusion of studies.

Parameter	Description
Population	General population, population with VitB12 deficiency and patients with dementia
Intervention/exposure	Elevated serum MMA levels
Comparison	Population with lower serum MMA levels or randomized control population or healthy control population
Outcome	Change in cognitive levels and in morbidity of dementia
Study design	Cross-sectional studies, randomized controlled studies and case-control studies

### Data Sources and Literature Search

In English retrieval, full-text search is carried out in PubMed, Web of science, EMBASE and ProQuest network databases with following Medical Subject Headings (MeSH) search terms: “cognition,” “methylmalonic acid,” “dementia,” and “Alzheimer’s disease,” and their synonyms. The full-text search of Chinese MeSH with the same meaning was carried out in WANFANG MED ONLINE, China National Knowledge Infrastructure (CNKI) and Chongqing VIP database. Manual retrieval was conducted to query professional journals, academic conference proceedings and the latest published academic journal materials if necessary. The retrieval time limit is from the start of the database to May 2022. The title and abstract of the articles were preliminarily screened by one author. Subsequently, two authors evaluated the full text of the articles. The full text of the candidate literature needs to record clear serum MMA concentration, and there is a statistical difference between the test group and the control group. The included studies should be cross-sectional studies, randomized controlled studies or case-control studies with people as the research object, and the outcome variables were reported in the form of mean and standard deviation. This meta-analysis excluded animal studies, case reports, self-reports and studies without control groups. When the two authors did not reach a consensus in the process of literature retrieval and evaluation, the third author would help to evaluate.

### Data Extraction and Synthesis

Data extracted from each ultimately selected study included the following: author name, year of publication, study design, country (region), age, sex, sample size, cognitive scores (mean and standard deviation) for treatment and control groups, disease type, and MMA levels in case group and control group.

### Quality Assessment

Two authors independently assessed the quality of the included studies. Cross-sectional studies were accessed using the Agency for Healthcare Research and Quality (AHRQ) assessment scale which includes 11 items, with a total of 11 scores. 0 ∼ 3 scores, 4 ∼ 7 scores, and 8 ∼ 11 scores are low, medium, and high quality in turn. The risk of bias of randomized controlled studies was assessed using the Cochrane handbook which had seven scores items each of which was classified as low risk, high risk, and unknown. The quality of included case-control studies was assessed using the Newcastle–Ottawa Scale with a total score of 9 and those with ≥6 were regarded as high-quality study. Any difference in the scores between the two authors was assessed by the third author.

### Data Analysis

RevMan 5.4.1 software was used for statistical analysis. All outcomes of included studies were calculated as standardized mean difference (SMD) or weighted mean difference (WMD) with 95% confidence interval (95% CI). *P* < 0.05 indicates that the difference was statistically significant. The heterogeneity among studies was assessed with *Q*-test and I^2^ statistic. I^2^ below 25% was considered as low heterogeneity, 25 ∼ 50% as medium heterogeneity, 50 ∼ 75% as high heterogeneity, and more than 75% was considered as very high heterogeneity. If *P* > 0.1 and *I*^2^ < 50%, it was considered that there was no statistical heterogeneity, and a fixed-effects model was used for meta-analysis; otherwise, a random-effects model was used for analysis. Subgroup analysis was used to explore the source of heterogeneity if necessary. The potential bias of included studies was analyzed by funnel chart. One by one exclusion method was used for sensitivity analysis.

## Results

### Search Results

**FIGURE 1:**
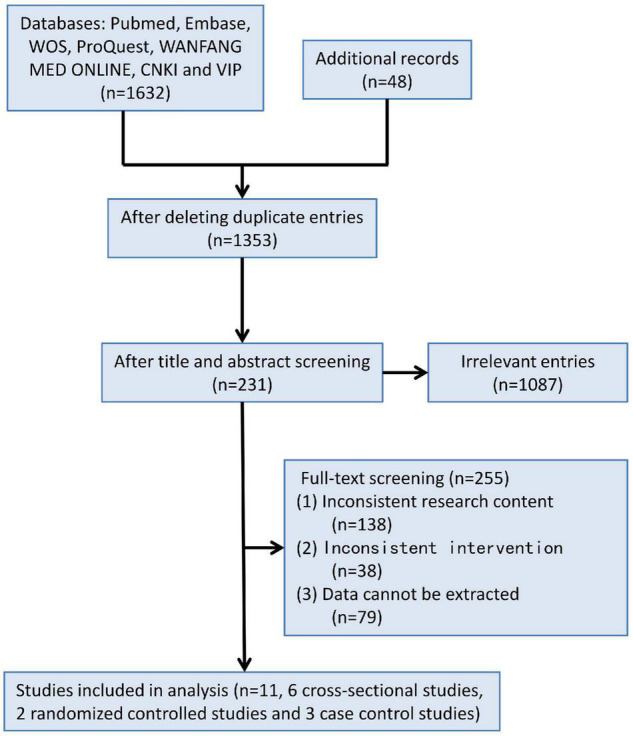
Flow diagram showing the study selection process.

1,680 records were retrieved according to the inclusion and exclusion criteria above. Through duplicate check, study design screening, abstract screening and full-text screening, 1,669 records were eliminated, and finally 11 studies were selected for meta-analysis, including six cross-sectional studies, two randomized controlled studies, and three case-control studies (Figure 1), with a total of 16,533 subjects.

**FIGURE 2:**
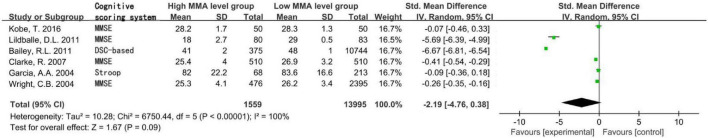
Forest plot of the association between MMA and cognition in the six cross-sectional studies.

Except for one of the studies involving children aged 6–11 months, the subjects in the other studies were adults aged ≥19 years, and most of them were elderly. All of the above study populations were from European and North American countries except one study from Nepal. The mean and standard deviation of cognitive score or plasma MMA level could be extracted from the 11 studies, and the cognitive scoring systems used in the included cross-sectional and randomized controlled studies were not identical (Table 2). The quality of the included study is of medium or high level (Tables 3, 4 and Figure 2).

**TABLE 2:** General characteristics of the included 11 studies.

Author (year)	Area/Country	Study design	Age	Sample size (female)	Cognitive scoring system/Disease
Kobe et al. (11)	Berlin and Frankfurt, Germany	Cross-sectional study	50–80 years	100 (52)	MMSE
Lildballe et al. (12)	Oxfordshire, United Kingdom	Cross-sectional study	≥75 years	163 (127)	MMSE
Bailey et al. (13)	United States	Cross-sectional study	≥19 years	11119 (5463)	DSC-based comprehensive scoring
Clarke et al. (14)	Oxford, United Kingdom	Cross-sectional study	≥65 years	1020 (?)	MMSE
Garcia et al. (15)	Ontario, Canada	Cross-sectional study	≥65 years	281 (?)	Stroop
Wright et al. (16)	Manhattan, New York, United States	Cross-sectional study	≥40 years	2871 (?)	MMSE
Strand et al. (17)	Bhaktapur, Nepal	Randomized controlled study	6–11 months	572 (?)	Bayley-III
Eussen et al. (18)	Netherlands	Randomized controlled study	≥70 years	100 (?)	Stroop
Miller et al. (19)	United States	Case-control study	Patients: 79 ± 7 years Control: 75 ± 6 years	54 (33)	Alzheimer’s disease
Nilsson et al. (20)	Sweden	Case-control study	Patients: 77.3 ± 8.6 years Control: 76.1 ± 8.0 years	130 (?)	Dementia
Lehmann et al. (21)	Sweden	Case-control study	71.5 ± 8.8 years	123 (73)	Senile dementia of the Alzheimer type

*?, unkown; MMSE, Mini-mental State Examination; DSC, Digit Symbol-Coding test scale; Stroop, Stroop neuropsychological screening scale.*

**TABLE 3:** Quality assessment of included 6 cross-sectional studies with Agency for Healthcare Research and Quality (AHRQ) assessment tool.

Study	(1)	(2)	(3)	(4)	(5)	(6)	(7)	(8)	(9)	(10)	(11)	Total score
(11)	Yes	Yes	No	UC	UC	Yes	Yes	Yes	Yes	Yes	No	7
(12)	Yes	Yes	Yes	Yes	UC	Yes	Yes	Yes	Yes	UC	No	8
(13)	Yes	Yes	Yes	UC	UC	Yes	Yes	Yes	Yes	Yes	No	8
(14)	Yes	Yes	Yes	No	UC	Yes	Yes	Yes	Yes	Yes	No	8
(15)	Yes	Yes	Yes	Yes	UC	Yes	Yes	Yes	No	Yes	No	8
(16)	Yes	Yes	Yes	Yes	UC	Yes	No	Yes	No	Yes	No	8

*UC, Unclear.*

*(1) Define the source of information (survey, record review).*

*(2) List inclusion and exclusion criteria for exposed and unexposed subjects (cases and controls) or refer to previous publications.*

*(3) Indicate time period used for identifying patients.*

*(4) Indicate whether or not subjects were consecutive if not population-based.*

*(5) Indicate if evaluators of subjective components of study were masked to other aspects of the status of the participants.*

*(6) Describe any assessments undertaken for quality assurance purposes (e.g., test/retest of primary outcome measurements).*

*(7) Explain any patient exclusions from analysis.*

*(8) Describe how confounding was assessed and/or controlled.*

*(9) If applicable, explain how missing data were handled in the analysis.*

*(10) Summarize patient response rates and completeness of data collection.*

*(11) Clarify what follow-up, if any, was expected and the percentage of patients for which incomplete data or follow-up was obtained.*

**TABLE 4:** Quality assessment of included 3 case-control studies with Newcastle–Ottawa Scale.

Study	Selection				Comparability		Exposure			Total score
	1	2	3	4	1	2	1	2	3	
(19)	*	*	*	*	*	*	*	*	*	9
(20)	*	*	*	*	*	–	*	*	*	8
(21)	*	*	–	*	–	–	*	*	*	6

–, no score. *, one score.

### Association Between Methylmalonic Acid and Cognition in Cross-Sectional Studies of the General Population

**FIGURE 3:**
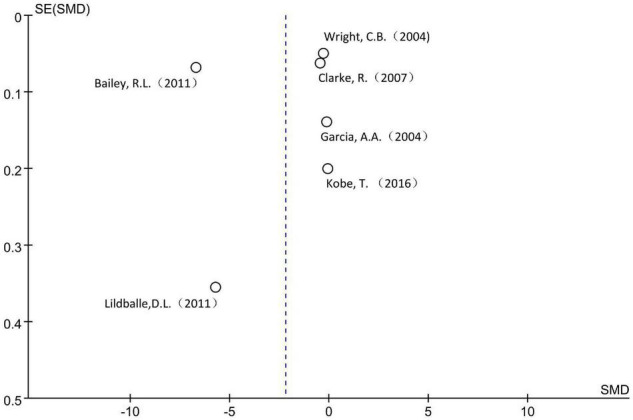
Funnel plot of the six included cross-sectional studies.

A total of 15,554 subjects were enrolled from the six cross-sectional studies, with an age distribution of ≥19 years. Random effect model was applied due to the statistical heterogeneity among the included studies (*I*^2^ = 100%). Meta-analysis showed that increased MMA level was not associated with decreased cognitive level in the general population without intervention, the difference was not statistically significant [SMD = −2.19, 95% CI (−4.76 ∼ 0.38), *Z* = 1.67, *P* = 0.09; Figure 2]. Cochrane funnel chart was used to visually evaluate the bias of the studies. The funnel chart showed incomplete symmetry suggesting that bias may exist (Figure 3). In the sensitivity analysis with one-by-one exclusion method, the results were: SMD = −1.16, 95% CI (−1.82 ∼−0.49), *Z* = 3.39, *P* = 0.0007, after removing the study of Bailey, R. L., etc. The results remained unchanged when other studies were removed one by one.

### Association Between Methylmalonic Acid and Cognition in Randomized Controlled Studies of Population Supplemented With Vitamin B12

**FIGURE 4:**
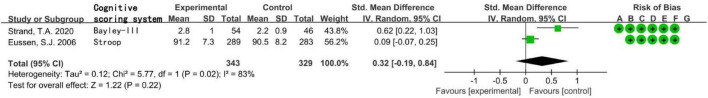
Forest plot of the association between MMA and cognition in the two randomized controlled studies.

The total number of subjects in the two randomized controlled studies was 672, including children aged 6–11 months in one study and elderly people over 70 years in the other study. The statistical heterogeneity I^2^ of the two studies was 83%, so the random effect model was used. Meta-analysis showed that in population supplemented with VitB12, the increase of MMA level was not related to the decrease of cognitive level, the difference was not statistically significant [SMD = 0.32, 95% CI (−0.19 ∼ 0.84), *Z* = 1.22, *P* = 0.22; Figure 4]. In the sensitivity analysis, the difference became statistically significant after removing the research of Eussen, S. J., etc. [SMD = 0.62, 95% CI (0.22 ∼ 1.03), *Z* = 3.03, *P* = 0.002]. The results remained unchanged after removing another study.

### Association of Methylmalonic Acid With Cognition in Case-Control Studies of Dementia Diseases

**FIGURE 5:**
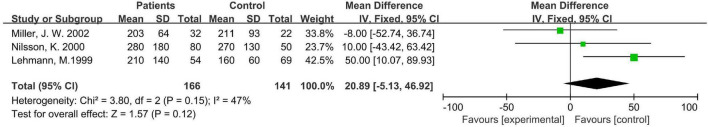
Forest plot of the association between MMA and dementia in the three case-control studies.

Three hundred seven subjects in the three case-control studies were included in the analysis, all of whom were elderly. The statistical heterogeneity I^2^ of the three studies was 47%, so the fixed effect model was used for analysis. The results showed that there was no significant difference in the level of MMA between the disease group and the healthy control group [WMD = 20.89, 95% CI (−5.13 ∼ 46.92), *Z* = 1.57, *P* = 0.12; Figure 5]. In the sensitivity analysis, when the research of Miller J. W., etc., was removed, the difference became significant [WMD = 35.66, 95% CI (3.68 ∼ 67.65), *Z* = 2.19, *P* = 0.03]. The results remained unchanged after removing the other two studies one by one. Sensitivity analysis showed that the stability of the above three meta-analysis was not sufficient.

## Discussion

Vitamin B12 plays an important role in the development and functional maintenance of the nervous system, and its strong correlation with cognitive function has also been confirmed in most studies. However, the metabolic regulation pathways of human body are staggered and complex, a nutrient deficiency can cause a variety of dysfunction, and a dysfunction can also be caused by different nutrients or intermediate abnormal metabolites. VitB12 is known to play a role in the form of coenzyme in human body, and its lack can cause abnormal accumulation of intermediate metabolites. The deep-seated causes of cognitive impairment are still unknown, and the correlation or even causality between the above metabolites and the formation of cognitive impairment cannot be ruled out.

A large-scale study based on the general population pointed out that the concentration of serum MMA began to rise at the age of 18–20, and this state will continue with age (22). Therefore, it is of great significance to explore the biological function and health impact of this metabolite. In this study, the relationship between MMA and cognition was comprehensively evaluated by meta-analysis for the first time. The final analysis results of this study showed that the increase of MMA serum concentration was not related to the decline of cognitive level, whether in the general non-intervention population or the population supplemented with VitB12, and there was no significant increase in MMA level in dementia diseases characterized by severe cognitive impairment.

Some studies suggested that MMA is not related to cognition. De Lau et al. observed no significant correlation between MMA and cognitive decline rate during follow-up (23). In another study, although low choline or betaine or low VitB12 or high MMA interacted significantly with cognitive performance, high concentration of MMA was not associated with decreased cognitive level (8). The same controversy exists in methylmalonic acidemia, which is characterized by increased MMA levels. Psychomotor retardation and intellectual disability are one of the common clinical manifestations of this disease. However, some studies have shown that the cognitive level of patients with methylmalonic acidemia fluctuates widely, and many patients have no cognitive impairment (24). Physical and cognitive outcomes were normal in a follow-up study of an asymptomatic cohort with persistent low to moderate urinary MMA elevation (25).

It should be pointed out that the result of this meta-analysis is inconsistent with our intuitive feeling during previous literature retrieval, because most of the relevant studies we encountered concluded that MMA is associated with the decline of cognitive level. In the meta-analysis of six cross-sectional studies in this study, two thirds studies confirmed their correlation. In a study of children aged 3–16, the increase of MMA was associated with the decrease of cognitive score (2). The deterioration of overall cognition of people with higher MMA concentration is accelerated, and the doubling of MMA concentration is related to the acceleration of cognitive decline by about 60% (23). MMA is negatively correlated with global cognition and episodic memory (26). High levels of MMA in community-based studies were not associated with brain volume loss but with cognitive impairment (27). Epidemiological studies have shown that high levels of MMA in circulatory system are associated with decreased cognitive function. In a longitudinal study conducted in the United Kingdom, the concentration of serum MMA increased from 0.25 to 0.50 μmol/L is associated with a 50% accelerated decline in cognitive level (28). In a multivariate analysis, a mixed effect model was used to test the association between each biochemical marker and cognition, and only MMA was associated with lower global cognitive score. In the 6-year follow up, the higher the MMA concentration, the faster the cognitive decline (29). Two other studies on the elderly in the community found that the increase of MMA concentration was related to cognitive impairment, but not to plasma VitB12 (30). High serum MMA is common in the elderly and negatively correlated with motor and cognitive performance (31). Increased MMA has also been confirmed to be associated with cognitive impairment in elderly stroke survivors (32). Some studies have concluded that the correlation between serum MMA and tHcy and cognitive impairment is stronger than VitB12 although the relationship between VitB12 and cognition is well known by researchers (33).

Increased MMA levels are also associated with other neurological diseases (34). The occurrence of high MMA (>360 nmol/L) in dementia patients was significantly higher than that in the control group (35). VitB12 deficiency defined by increased MMA levels is associated with an increased risk of dementia diagnosis (36). Studies have shown that the increase of MMA and tHcy, rather than the level of plasma VitB12, is related to pathologically confirmed Alzheimer’s disease (30). In a case-control study of Alzheimer’s disease, MMA levels were higher in the case group than in the control group (*P* = 0.027) (37). While the proportion of cognitive impairment in the population with increased plasma MMA is increased, the prevalence of depression is also quite high (38). In cblC disease characterized by increased MMA levels, some of the detected protein changes were similar to those in Alzheimer’s disease (39).

Methylmalonic acid may have neurotoxicity, which is supported by its tissue distribution characteristics. Cerebrospinal fluid MMA correlated with serum MMA with an r of 0.69, *P* < 0.0001, and mean cerebrospinal fluid MMA was 170% of mean serum measurement. The levels of several similar analytes, such as VitB12, holotranscobalamin and tHcy, in cerebrospinal fluid were much lower than in serum (40). Study has shown that higher substantia nigra echogenic area was associated with higher plasma concentrations of MMA (41). High concentrations of MMA are also associated with more severe white matter lesions (WML), although this association is statistically significant only in periventricular WML (42).

Funnel plot shows that there may be serious bias in this meta-analysis. In the sensitivity analysis, after removing one of the studies of the three meta-analyses separately, statistically significant differences in cognitive scores were detected, indicating that the stability of the results of this study is not sufficient. This may be related to the special data extraction method. Up to now, there are few population cohort or case-control studies with MMA as the risk factor alone, so the data of this study were mainly extracted from the researches on the relationship between B vitamins and their multiple markers and diseases, which made the grouping criteria of the original subjects by MMA level not very consistent, this may make the data unable to reflect the real situation to a certain extent and affect the accuracy of the results.

To sum up, the results of this meta-analysis show that elevated MMA is not associated with cognitive decline. Available trials have limitation in the original data. Further well-designed large sample prospective studies and basic mechanism researches are needed so as to provide a solid and reliable basis for the prevention and treatment of cognitive diseases.

## Data Availability Statement

The original contributions presented in this study are included in the article/supplementary material, further inquiries can be directed to the corresponding author.

## Author Contributions

WL and CW contributed to conception and design of the study. CW and YZ search the databases and performed the statistical analysis. CW, YZ, YY, and JS did the screening of abstracts and full manuscript for inclusion. CW and JS wrote sections of the manuscript. All authors contributed to manuscript revision, read, and approved the submitted version.

## Conflict of Interest

The authors declare that the research was conducted in the absence of any commercial or financial relationships that could be construed as a potential conflict of interest.

## Publisher’s Note

All claims expressed in this article are solely those of the authors and do not necessarily represent those of their affiliated organizations, or those of the publisher, the editors and the reviewers. Any product that may be evaluated in this article, or claim that may be made by its manufacturer, is not guaranteed or endorsed by the publisher.
